# Temporal Association Rule Mining: Race-Based Patterns of Treatment-Adverse Events in Breast Cancer Patients Using SEER–Medicare Dataset

**DOI:** 10.3390/biomedicines12061213

**Published:** 2024-05-29

**Authors:** Nabil Adam, Robert Wieder

**Affiliations:** 1 Phalcon, LLC., Manhasset, NY 11030, USA; n.adam@phalconllc.com; 2 Rutgers University, Newark Campus, Newark, NJ 07102, USA; 3 Rutgers New Jersey Medical School, Newark, NJ 07103, USA; 4 Rutgers Cancer Institute of New Jersey, Newark, NJ 07103, USA

**Keywords:** temporal association rule mining, breast cancer, adverse events, racial disparities, SEER–Medicare linked dataset

## Abstract

PURPOSE: Disparities in the screening, treatment, and survival of African American (AA) patients with breast cancer extend to adverse events experienced with systemic therapy. However, data are limited and difficult to obtain. We addressed this challenge by applying temporal association rule (TAR) mining using the SEER–Medicare dataset for differences in the association of specific adverse events (AEs) and treatments (TRs) for breast cancer between AA and White women. We considered two categories of cancer care providers and settings: practitioners providing care in the outpatient units of hospitals and institutions and private practitioners providing care in their offices. PATIENTS AN METHODS: We considered women enrolled in the Medicare fee-for-service option at age 65 who qualified by age and not disability, who were diagnosed with breast cancer with attributed patient factors of age and race, marital status, comorbidities, prior malignancies, prior therapy, disease factors of stage, grade, and ER/PR and Her2 status and laterality. We included 141 HCPCS drug J codes for chemotherapy, biotherapy, and hormone therapy drugs, which we consolidated into 46 mechanistic categories and generated AE data. We consolidated AEs from ICD9 codes into 18 categories associated with breast cancer therapy. We applied TAR mining to determine associations between the 46 TR and 18 AE categories in the context of the patient categories outlined. We applied the spark.mllib implementation of the FPGrowth algorithm, a parallel version called PFP. We considered differences of at least one unit of lift as significant between groups. The model’s results demonstrated a high overlap between the model’s identified TR-AEs associated set and the actual set. RESULTS: Our results demonstrate that specific TR/AE associations are highly dependent on race, stage, and venue of care administration. CONCLUSIONS: Our data demonstrate the usefulness of this approach in identifying differences in the associations between TRs and AEs in different populations and serve as a reference for predicting the likelihood of AEs in different patient populations treated for breast cancer. Our novel approach using unsupervised learning enables the discovery of association rules while paying special attention to temporal information, resulting in greater predictive and descriptive power as a patient’s health and life status change over time.

## 1. Introduction

African American (AA) women have significantly higher mortality rates from breast cancer (BC) than White (W) women with the same disease and patient variables despite having a lower incidence of disease [1,2,3,4,5,6]. Genetic [7] and epigenetic [8] differences in cancer cells [9] and genetic, immune, and inflammatory differences and vitamin D deficiency in the tumor microenvironment [8,10,11] contribute to more aggressive cancer characteristics and, in combination with extensive and well-documented socioeconomic factors [12,13], collectively generate a worse prognosis.

Treatment (TR) quantity and quality frequently differ between the two races due to several social factors [13,14]. In addition, TR-induced AEs impose significant obstacles to tolerating therapy, the quality of life, and the ability to administer adequate therapy, which affects outcomes [15]. While several studies [16,17] suggest that AA race is a risk factor for TR-associated AEs, data showing differences are limited to the effects of a few cytotoxins and the impact of comorbidities [16,17,18,19,20,21,22,23,24,25]. Several studies show no statistical differences between the two groups [6,18].

The limitations of published investigations prevent the formulation of effective conclusions that can be applied to specific circumstances of medical practice to address the severity of the problem. Most studies have relatively small sample sizes, are not systematic, use population-averaged analyses, and often fail to detect differences among the races [26]. Circumstance-specific scenarios that occur in the real world, which depend on patient variables, including age, comorbid conditions, and prior therapy, a spectrum of cancer-specific variables and TR-associated variables, and their temporal associations, often reveal the specific AEs that otherwise fade into the background noise of population-averaged studies. Accurate and significant data are unavailable for most of these variables and can only be obtained by analyzing large databases. We use the SEER–Medicare linked dataset and apply temporal association rule (TAR) mining to discover temporal associations between BC TR and AEs and contrast them between AA and W BC patients considering two outpatient cancer care providers and venues: caregivers in hospital and healthcare facilities and private practitioners and non-institutional providers in office practices.

Regression analysis, which aims at understanding the association between the treatments, e.g., $X_{1}$ and $X_{2}$, and the outcome, Y, might show, for example, how $X_{2}$ relates to Y when $X_{1}$ is fixed. Causal mediation analysis [27] helps identify intermediate variables that might lie in the causal pathway between the treatment, $X_{1}$ and the outcome, Y. In Gotlieb et al. [28], applied causal mediation analysis to determine the SpO2–hemoglobin oxygen saturation discrepancy as a mediator of the effect of race and ethnicity [27]. Collaborative filtering is the most successful and widely applied technique in designing recommender systems that provide associations between users and items. Collaborative filtering, however, suffers from the data sparsity problem [29]. To overcome the data sparsity problem, data mining techniques such as clustering, classification, Singular Value Decomposition, and association rule mining have been applied to recommender systems.

Association rule mining is a data-driven process where a pattern is derived based on the available data while making no assumption about the extracted pattern. Further, in our case, the temporal aspect of the data is a critical factor; we therefore adopted the TAR mining technique. Our novel approach, an unsupervised machine learning, enables the discovery of rules of association embedded in the data while paying special attention to temporal information, resulting in greater predictive and descriptive power since patients’ health status and comorbidities, as well as social features that affect health, vary over time in real life. This novel approach was especially effective when, as in our case, dealing with high-dimensional data with deep structures, thus helping to overcome the challenges of predicting associations and race- and venue-based disparities between breast cancer treatment and adverse events for the first time.

## 2. Methods

### 2.1. Study Data: SEER–Medicare Linked (S-M) Dataset

**Figure 1:**
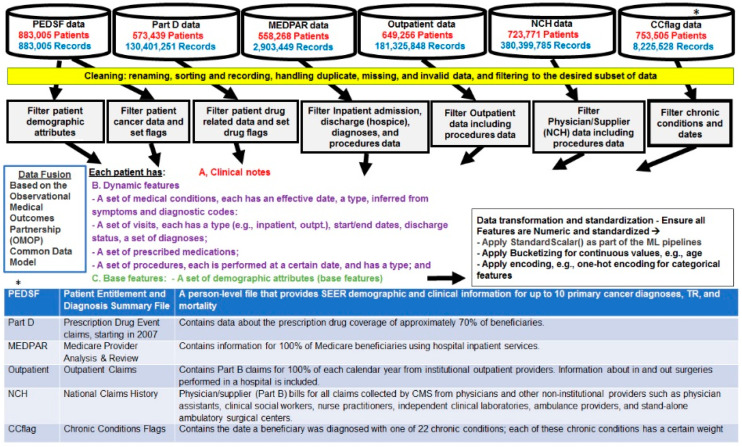
Pre-processing of the breast cancer patient data. *PEDSF, Part D, MEDPAR, Outpatient, NCH and CCflag data files definitions are defined, noted by * in the lower part of the figure.

The study data are longitudinal and large in volume, with hundreds of thousands of patients and hundreds of millions of related records (Figure 1). The dataset [30] links two datasets, each large and complex on its own: the National Cancer Institute’s Surveillance, Epidemiology, and End Results (SEER) and the Centers for Medicare & Medicaid Services (CMS) Medicare data. The SEER, a major source to assess cancer care and outcomes in the US, includes cancer incidence data for approximately 26% of the US population in various regions. The CMS Medicare data are a rich source of information about cancer care and outcomes in the older (65 years and older) population.

We fused the data in the various files based on the Observational Medical Outcomes Partnership (OMOP) common data model. This enabled us to transform data contained within those disparate FILES into a common format (data model), as well as a common representation (terminologies, vocabularies, and coding schemes).

### 2.2. Inclusion Criteria

In our study, we included women with the diagnosis of stages I, II, III, and IV BC who have not had any other malignancy history except non-melanoma skin and eyelid cancer, as a standard in NCI clinical trials [31]. We included all comorbidities for each patient recorded at every visit from the date of diagnosis, all prior TRs, delineated age, race, marital status, BC stage, grade, hormonal, and Her2 status, and laterality. Analyzing only patients who had these inclusion characteristics recorded permitted comparison of defined patient groups. We grouped patients into stages I–III and stage IV to analyze treatments characteristic of the two-stage grouping according to practice criteria. We included only patients who were enrolled in both Parts A and B of Medicare and not HMO, with a diagnosis of BC and whose age at enrollment was 65 or older, to restrict the analysis to patients who represented the general Medicare patient population. Thus, they were qualified by age and not disability, which would affect their treatments and AEs. The enrolled population was, therefore, skewed by age and only represented an older subset of the BC patient population.

For this study, we included claims whose CLM_TYPE = 40, 41, 42, or 71 and/or whose OPSRVTYP = 3. Such a claim was classified as (1) Private Practice Office (PPO) care provider if PLCSRVC = 11, or (2) Institutional Outpatient (Inst. OP) care providers, if its FAC_TYPE = 1 or 2 and/or PLCSRVC = 13, 22, 26, 31, 32, 50, 71, 72 (Table 1).

We generated a dataset of patients who were identified by the above criteria as cared for by physicians working in an Inst. OP facility. There were 242,598 BC patients, 21,148,628 visits, and 2,601,753 entries. In addition, we generated a dataset from the PPO files that included 236,198 BC patients, 8,993,967 visits, and 90,851,698 entries.

We included all patients’ ICD-9 and HCPCS codes. We annotated 141 HCPCS drug J codes: 82 chemotherapy drugs, 49 biotherapy drugs, and 10 hormone therapy drugs. The list included licensed drugs used or investigated in clinical trials for BC. We consolidated the drugs into 46 mechanistic categories and one no-TR category. We classified categories by mechanisms of action, such as alkylating agents, antimetabolites, anthracyclines, antimicrotubule agents, antiemetics, growth factors, monoclonal antibodies and small molecules, and hormone receptor inhibitors, for example, to generate the AE data. We consolidated related AEs into categories to enable a more comprehensive analysis of the variables associated with their occurrence. We included all ICD codes of potential AEs in a category, regardless of rarity, to ensure we did not miss any AEs. The broad categories represented general categories of adverse cancer TR events outlined in the literature and included 0) no-AE and ICD codes associated with 18 categories of AEs, outlined in the algorithm below. We excluded categories such as chronic infections, HIV, positive TB tests, contacts, carriers, procedural, traumatic, neonatal or infantile infections, childhood, pregnancy, or parturition.

Patient characteristics in the two stages and the two races in the two venues are outlined in Table 2. Patient numbers and distributions were similar in the two venues. Approximately 95% of W patients had stage 1-III BC, 5% had stage IV BC, 91.5% of AA patients had stage I–III BC, and 8.5% had stage IV BC in both venues. Patients in stages I–III were approximately 92.6% W and 7.4% AA in both venues, while stage IV patients were approximately 88.5% W and 11.5% AA in both venues. The mean ages of the patients in each category were similar in both venues. AA patients were younger than W patients in both stage groups and venues, and stage IV patients in all groups were older than stage I–III patients. The differences were statistically significant, but actual differences were minimal. The general mean age averages were in the mid-70s, indicative of the dataset population enrolled in Medicare at age 65 for age eligibility only. Of note, data with this population do not necessarily reflect the general population of women with BC. Comorbidities in the stage I–III population were statistically significantly higher in AA patients than W patients in both venues, but they were lower and similar in stage IV patients.

### 2.3. Data Cleaning and Standardization

We removed duplicate records in the dataset and included patients with valid patient id, cancer type (breast, or breast plus skin, or eyelid), stage (I–III, IV), sex, and date of diagnosis (month/year; in case of a missing day of the month, we assumed the first day of the month). We included only valid diagnoses, procedures, or HCPCS codes at a given visit. We applied the Melt transformation to convert a visit record (containing a mix of valid and missing codes) from wide format to stacked/long form, thus being able to delete invalid codes while keeping only valid ones for each visit. We performed data transformation and standardization, ensuring all features were numeric and standardized.

### 2.4. Association Rule Mining

Association discovery is one of the most common data mining techniques for extracting interesting knowledge and identifying hidden patterns from large datasets. Association rule mining has been successfully applied in various areas, including medicine [32,33] and biomedicine [34], traffic safety [35], and energy [36].

Given a dataset, $D=\left\{Trans_{1},Trans_{2},\ldots ,\;Trans_{m}\right)$, with m temporal transactions, and $I=\{i_{1},\;i_{2},\;\ldots ,i_{k}\}$ is an itemset that contains k variables. An association rule between X and Y implies X⊂Y, and the general form of the rule is X→Y, which can be read as “if antecedent X is true then consequent Y must be true”, where X and Y are sets of different items (itemsets) in a dataset and satisfy X∩Y = Ø [36,37]. This rule indicates that the X set will likely occur whenever the Y set occurs. In our case, X and Y represent TRs, AEs, disease, e.g., stage I, II, III, or IV, ER/PR/Her2 status, or patient characteristics, e.g., age. For example, TR4, Age_grp2 → AE3, indicates the association of category 4 TR and patients belonging to age group 2 on the one hand with the category 3 AE.

**Definition 1.**  *Support(*X*) is the probability that transaction* X *appears in* D*, and =* $\frac{count(X)}{|D|\;}$.

**Definition 2.**  *Support(*X→Y*) is the probability that transactions* X and Y *appear together in* D*, and = Support(X ∪ Y). It indicates how often this rule is applicable to D.*

**Definition 3.**  *Confidence(*X→Y*) is the probability that transactions* Y *appear in* D*, given that* X *appears in* D*, and is given by* $\frac{P\left(X\cap Y\right)}{P\left(X\right)}$*. To ensure the accuracy and relevance of the mined rules, lift is a third measure of a rule’s relevance.*

**Definition 4.**  *Lift(*X→Y*) measures the degree of correlation between X and Y: independent (=1), positively related (>1), and negatively related (<1). It is given by* $\frac{Support\left(X⋃Y\right)}{P\left(Y\right)*P\left(Y\right)}=\frac{P\left(X\cap Y\right)}{P\left(X\right)P(Y)}$* . It is worth noting that, in our text, a rule,* X→Y*, is not interesting if* X  *and* Y *are approximately independent. A Lift(*X→Y*) > 1 means that the occurrence of* Y *implies the occurrence of* X*. The larger the Lift value, the stronger Y positively correlates with* X*. A lift value of 2 means that the number of examples of this rule is twice what is expected under independence. Thus, a patient who has received treatment X is twice as likely to experience adverse event Y than the regular patient and the reverse is true since Lift is symmetric [38].*

**Figure 2:**
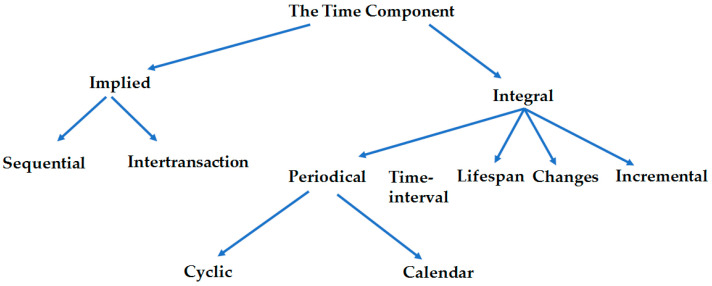
TARs Taxonomy.

Apriori [37] and FPGrowth [39] are the most common algorithms for mining frequent item sets. FP-Growth, which represents the database in the form of a tree called a frequent pattern tree (FP tree), is an improvement to the Apriori method. Advantages of the FPGrowh method include generating a frequent pattern without the need for candidate generation and a reduced search for frequent item sets. Given the large volume and complex aspects of our study data, we applied the spark.mllib implementation of the FPGrowth, a parallel version called PFP [40], which distributes the work of growing FP-trees based on the suffixes of transactions, resulting in a scalable implementation. TAR mining methods, which deal with datasets with temporal information, have been applied in medicine and healthcare [41,42,43,44,45,46,47]. Segura-Delgado et al. [48] presented an overview of the various TAR mining methods and proposed a taxonomy to classify these methods based on the time component (Figure 2). According to their taxonomy, time could be an implied or integral component. Methods belonging to the implied category use temporally ordered datasets to discover temporal constraints. In the case of the integral component, methods integrate the time variable as a data attribute and analyze the temporal aspects where the rules occur.

In our case, cancer TR or an AE is associated with a timestamp; a TAR can capture their temporal progression. A temporal dataset can be categorized into one of three categories. Datasets belonging to category 1 have each transaction associated with a timestamp [49,50]. A transaction in a dataset belonging to category 2 is associated with a time interval [51,52]. In a temporal dataset belonging to category 3, a transaction is associated with a time stamp, and the same item can be associated with two different time stamps [32,53]. Consider, for example, AE 4; a patient may experience AE 4 at a time $t_{1},\;$ while another patient (or even the same patient) may experience the same AE at a time $t_{2}.$ The S-M dataset belongs to category 3.

Proposed methods dealing with TAR mining [32,37,53,54] typically include two phases: phase 1 involves mining frequent item sets from the temporal dataset, and in phase 2, the discovered frequent item sets are used to generate TARs. The results of the first phase can be obtained by applying the FPGrowth; the method introduced in [37] is always applied to the second phase. This method, however, requires the finding of all proper subsets of each frequent itemset; in case of a large number of frequent item sets discovered from the dataset, the second phase is time-consuming [43].

### 2.5. Discovery of TR-AE Patterns

In our dataset, visits represent successive time points and there is a dimension along which they are ordered. Therefore, we should not treat each visit as independent and predict the AE based on, for example, a particular time of the visit regardless of what happened earlier. The existence of order enables us to exploit association relationships across time and, for example, predict the AE status at a given visit based on not only the TRs administered at this visit but also TRs at earlier visits.

One approach is to discover TARs from a time-ordered sequence of events. A TAR, R, indicates the occurrence of an item set, Y likely follows the occurrence of item set X after a time gap, Δ with a certain probability would be of the form: $X\to \left(\Delta \right)Y$, where X and Y are two frequent item sets $\left(X\cap Y=Ø\right)$ [43]. In Nguyen et al. [43], used the following measures to evaluate the quality of temporal rules [43]: Direction support of a TAR, R$\;dirsup\left(R\right)=|\{P_{i}|P_{i}\in Pidset\left(X\cup Y\right)\wedge \min\left(Y,P_{i}\right)-\max\left(X,P_{i}\right)\geq \Delta |/|D|$, where |D| is the number of patients in the dataset. The above method, however, does not consider a rule’s lift, which measures the degree of correlation between the antecedent and the consequent.

Our proposed approach explicitly takes into account the temporal information of our dataset and, at the same time, considers the confidence, support, and lift of an induced rule. Thus, we view the dataset in terms of “patient-sequence”, i.e., a sequence of patient id, visit id, timestamp, and a list of TR and AE codes that are included. Below is an example of a patient sequence:

Patient-visit ID       TR, AE sequence

20071821903-20019      TR3, AE1

We generated from the current dataset a new dataset where visits with the same patient id-visit ID are ordered according to their timestamps and arranged in a single sequence, patient-sequence, where each patient-sequence is a sequence of codes.

A sequence of item sets $\left(X_{1},X_{2},\ldots ,X_{n}\right)$ is considered a subsequence of another sequence $\left(Y_{1},Y_{2},\ldots ,Y_{m}\right)$ with n≤m  if $\left(X_{1}\subseteq Y_{i_{1}},{X_{2}\subseteq Y_{i_{2}}X}_{2},\;\ldots ,X_{n}\subseteq Y_{i_{n}}\right)\;\mathrm{and}\;i_{1}<i_{2}<,\ldots ,<i\_n$. Thus, the support of a sequence, S (Support(S)), is defined as the frequency with which a subsequence of the patient sequence is present in the dataset [48].

## 3. Experimental Design

### 3.1. Identifying TR-Associated AEs

To apply the proposed approach, we converted the dataset into a transaction dataset, where each transaction is a patient’s visit taking place on a given date, and items are (1) TRs administered to each patient on each visit and (2) AEs experienced by each patient on a given visit caused by this visit’s TRs or TRs administered within the past 21 days. We opted for 21 days because we were investigating acute AEs and wanted to ensure they included TR-induced thrombocytopenia, which may take that long or longer to recover [55]. Many AEs last much longer, and this approach excluded them. This time frame increased the likelihood that the AEs associated with a TR were not present at the time of the TR [56].

An acute AE of a given TR may occur within the same day the TR was administered or within several days after the administration of the TR. The distribution of a flagged AE entry temporally associates a specific AE category with a specific TR category within a specified period (Algorithm 1).
**Algorithm 1.** Treatment/Adverse Event Algorithm.**Input:** Acute_Period = 21 days **Output:** For Each Patient: (1) treatments administered on each visit that caused an adverse event on the day of the visit (AE_TR_List) or within 21 days prior to this visit (AE_Pre_TR_List); (2) adverse events experienced by each patient on a given visit caused by this visit’s treatments (AE_List) or treatments administered within the past 21 days (Pre_AE_List); (3) adverse event flag, for each entry of every visit, indicating the status of the adverse event caused by this entry treatment, AE_flag1, or caused by prior treatment, AE_flag2; and (4) treatments administered to each patient on each visit that caused no adverse event on the day of the visit (No_AE_TR_List), or within 21 days prior to this visit (No_AE_Pre_TR_List).**Initialization:**  AE_list1 = [1, 7, 8, 11–13, 15], AE_list2 = [2–6, 9, 10, 14, 16–18]           AE_TR_List = [], AE_Pre_TR_List = [],AE_List = [], Pre_AE_List = [],            Pre_V_AE_List = [], No_AE_Pre_TR_List = [], Pre_TR_List = []**For Each Patient****For Each Visit**Compute the Visit Time-stampOrder Visits. based on Visit’s Time-stamp (in ascending order)Compute Elapsed Time Between Each Consecutive Visit**For Each Entry within this Visit**AE_flag1 ← 0, AE_flag2 ← 0 (initialize corresponding AE flags)Convert Every HCPCS Code to Corresponding Treatment Category (0–46), TRConvert Every ICD Code to Corresponding Adverse Event Category(0–18), AE**For Each TR/AE combination**Append this AE to the AE_ListIf AE = 0: Append this TR to the No_AE_TR_ListIf AE belongs to AE_list1&Elapsed time since this AE occurred > Acute_Period:AE_flag1 ← 1, Append This TR to the AE_TR_ListIf AE belongs to AE_list1 or AE_list2 & time since this AE occurred > Acute_Period:**For Each Pre_TR > 0 in Pre_TR_list**If time since this Pre_TR administered > Acute_Period:AE_flag2 ← 1Append this TR to the AE_Pre_TR_list

AEs most commonly associated with chemotherapy in prior studies were included [10,15,55,56,57,58,59,60]. They were generated based on clinical events, where, in practice, the administration of TR can induce fever, weakness, malaise or disorientation, nausea, pulmonary embolus, edema, rash, or respiratory symptoms the same day. At the same time, the other AEs may take at least a day to manifest. The study documents acute events that extend to 21 days, a period chosen to include TR-induced thrombocytopenias that may take three or more weeks to resolve. Hence, the two sets of AEs we identified were (1) AE_set_1 = [AE 1, 7, 8, 11–13, 15], where the AE is said to occur if it takes place within 0–21 days from the day TR was administered, given that it did not occur 21 days earlier; (2) AE_set_2 = [AE 2–6, 9, 10, 14, 16–18], where the AE is said to occur if it takes place within 1–21 days from the day TR was administered, given that it did not occur 21 days earlier.

**Figure 3:**
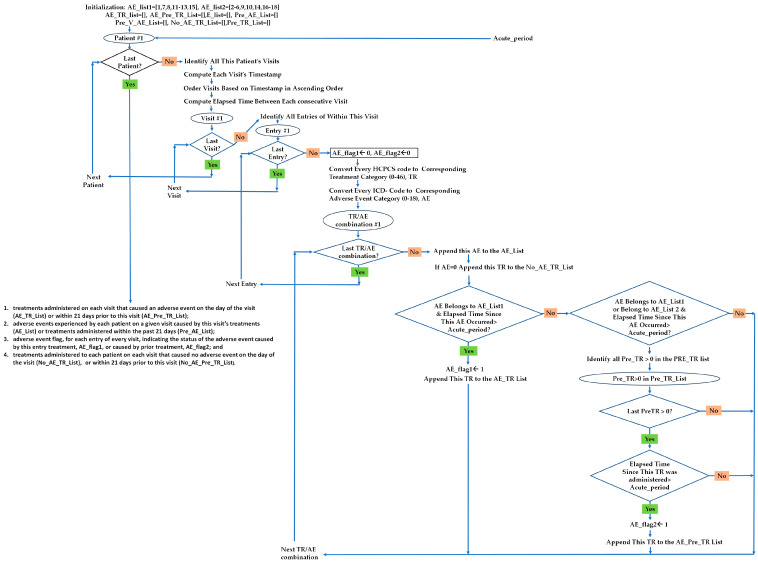
Algorithm for temporal association analysis.

A patient’s visit may have multiple entries (an entry for each diagnosis/HCPCS combination). For each entry associated with a given visit, we identified a set of AEs to account for the temporal aspect of the TR/AE, we adopted the period as 21 days, and for each patient, we identified each AE occurring as a result of one or more TRs administered within 21 days (referred to as Pretreatment); this is in addition to AEs occurring within the same day of TRs. We attributed the recordable AE flagged by the algorithm (a flowchart of the algorithm is depicted in Figure 3) to each TR category administered on the same day or within the 21 days defined by the algorithm since it is impossible to discern from the data which TR was responsible for it.

To reduce the heterogeneity of the patient population, we divided the patients into two groups. Group 1 included stage I–III patients and Group 2 included stage IV patients. We further divided each patient group into two subgroups: W and AA.

We applied the frequent pattern mining algorithm, FPGrowth, to our dataset. When applying the FPGrowth algorithm, a key problem is choosing a minimum support value to find interesting patterns. There is no straightforward way to determine the best minimum support threshold; it is usually performed by trial and error. Generally, a high minimum support value when the dataset is small ensures that the item sets are significant. However, a high minimum support value, e.g., 50%, when having a small dataset, e.g., only four patient entries, would result in item sets that are not interesting (only two entries in this case).

**Figure 4:**
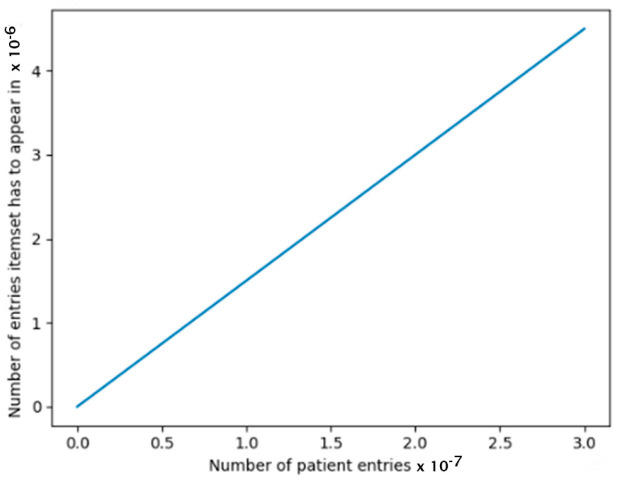
Minimum Support.

On the other hand, having a 50% minimum support value when we have 10,000 patient entries results in item sets having to appear in 5000 entries, which is too high a requirement, i.e., as the minimum support becomes larger, the less temporal frequent item sets will be generated. We want to be able to discover frequent code sets that are still large enough to represent the patient population. To determine the minimum support (minSupp) so that the threshold is higher when the item set size is small, and the threshold is low when the item set is larger, we adopted a minimum support value (minSpp) in the form of a step function:

$$\mathrm{minSupp}=\left\{\begin{array}{c} \;x<x_{0},\;\;\;\;\;\;\;\;\;\;\;\;\;\;\;\;\;\;e^{-a_{0}*x+b}+c\\ x_{0}\geq \;x\leq x_{1},\;\;\;\;\;\;\;\;\;\;\;\;\;e^{-a_{1}*x+b}+c\;\\ \ldots \\ x_{n-1}\geq \;x\leq x_{n},\;\;\;\;\;\;\;\;\;\;\;\;\;e^{-a_{1}*x+b}+c \end{array}\right.$$

where x is the number of patient entries, $a_{i}$, b, and c are constants. Based on our database size and number of patient entries, we set the values of $a_{i}$, b, and c to result in itemsets that are significant (Figure 4).

**Definition 5.**  *Valid TAR. Given a minimum confidence threshold λ ∈ [0, 1] and a minimum lift threshold, a rule R:* $X\to \left(\Delta \right)Y$*, is considered a valid TAR if conf(R) ≥ λ and lift(R) ≥ φ.*

To achieve high accuracy and potential usefulness of the identified rules for predicting the consequent in future data, we set the minimum confidence, λ, to 95% and the minimum lift, φ, to 2.0. Of note, different TRs/AEs associations are discovered at different lift values. Depending on the mix of the TRs, AEs might differ since these AEs reflect the effect of the set of TRs included at a specific value of the lift.

### 3.2. Validation

To validate the TAR Mining approach for identifying high correlations between TRs and AEs, we determined the actual rates of AEs with two drug categories administered in stage I–III Inst. OP scenarios. We recorded all AEs that occurred at least 1% of the time with each drug and listed them in order of frequency.

## 4. Results

### 4.1. TAR Mining Predicted TR/AE Associations

**Figure 5:**
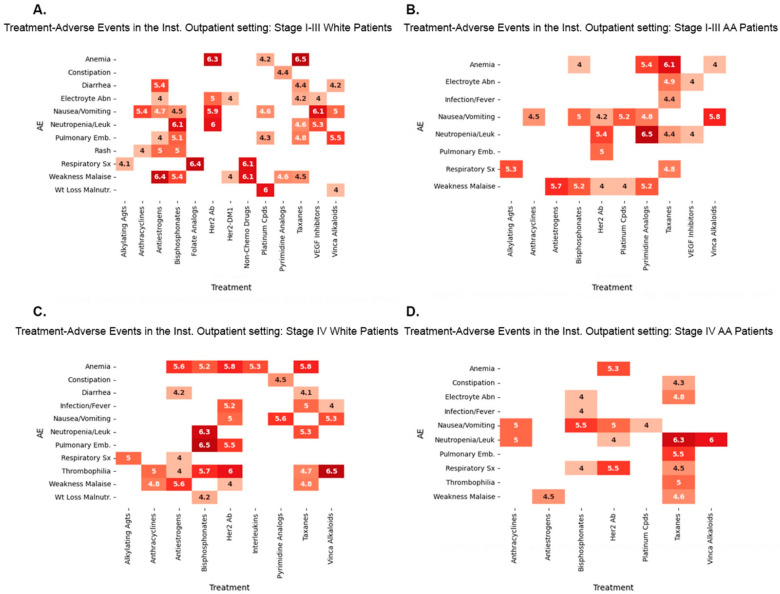
Association of TR and AE categories defined by lift (≥4) in the Institutional Outpatient setting for (**A**). Stage I–III White patients, (**B**). Stage I–III AA patients, (**C**). Stage IV White patients and (**D**). Stage IV AA patients. Lift 4–4.4 [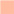], 4.5–4.9 [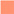], 5.0–5.4 [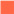], 5.5–5.9 [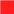], 6.0–6.4 [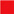], and 6.5–6.9 [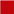].

**Figure 6:**
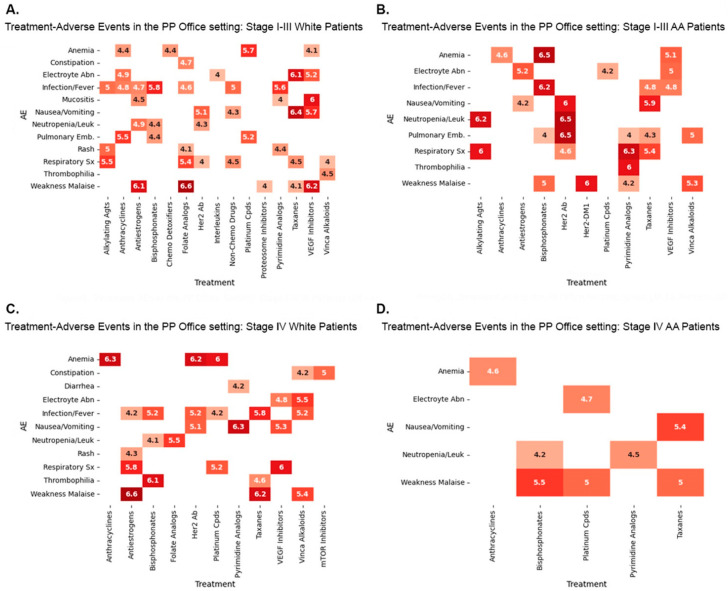
Association of TR and AE categories defined by lift (≥4) in the Private Practice (PP) Office setting for (**A**). Stage I–III White patients, (**B**). Stage I–III AA patients, (**C**). Stage IV White patients, and (**D**). Stage IV AA patients. Lift 4–4.4 [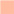], 4.5–4.9 [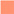], 5.0–5.4 [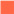], 5.5–5.9 [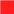], 6.0–6.4 [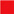], and 6.5–6.9 [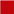].

Figure 5 and Figure 6 depict the highest lift associations, and Supplemental Tables S1–S4 list all the lift two or higher associations. We set the minimum significance at a lift of 2 to be at least one lift point above lift 1, indicating no association. We considered differences of at least one lift as significant in all our comparisons of TR/AE associations between W and AA patients. Some lift values varied when the temporal association threshold was changed from 2 to 4 (Tables S1–S4). All the results have a 95% minimum confidence level.

The administration of chemotherapy, biotherapy, and immunotherapy drugs to stage I–III breast cancer patients is associated with high-lift TR/AE associations that vary by venue of care and race. Multiple, diverse patterns of associations between TRs and AEs were evident with different drug categories. Some drugs, such as Her2 Ab, bisphosphonates, and pyrimidine analogs, result in different sets of associations that are greater by at least one lift in W than AA patients, greater in AA than W patients, and are also different from one another in the Inst. OP and) between the treatment and the AE.

Other high-lift AE associations with treatments such as taxanes, alkylating agents, anthracyclines, and VEGF inhibitors, for example, only occur in one race but are not evident in the other race in one treatment venue but have unique TR/AE associations by race in the other venue. Some TR/AE associations, such as those observed with folate analogs, Her2-DM1, and interleukins, are observed only in one racial category. Some TR/AE associations, for example, those found with antiestrogens and platinum compounds, may be present in different races and settings. Some TR/AE associations, such as those observed with vinca alkaloids and VEGF inhibitors, for example, may be the same for one race in both the Ins. OP setting, as well as the PPO settings.

Stage I–IV patients also experience race and venue-based preferential TR/AE associations, but unique associations in the four possible categories, as found with some stage I–III drugs, are not observed in stage IV scenarios. This phenomenon may be influenced by the relatively small number of values in each category. Race- and venue-dependent TR/Associations in all four combinations are observed with taxanes, Her2 Ab, bisphosphonates, antiestrogens, and platinum compounds. However, Stage I–IV patients have more specific TR/AE associations observed in the same race and in both venues, such as those occurring with taxanes, Her Ab, bisphosphonates, anthracyclines, antiestrogens, platinum compounds, and VEGF inhibitors, than stage I–III patients. Some TR/AE associations with some drugs, such as alkylating agents, pyrimidine analogs, vinca alkaloids, interleukins, VEGF Inhibitors, folate analogs, and mTOR inhibitors, for example, are only seen in one of the racial categories, either in one venue or both venues. The distributions in the two-stage categories highlight stark differences in high-incidence TR/AE associations that depend on race and the venue of care and suggest that different practices and TR approaches occur between the two racial groups in the two TR venues.

### 4.2. Validation of TR-Associated AEs

As shown in Table 3, there is a high degree of overlap between the model’s predicted TR/AE associations and actual TR/AE associations; albeit, the order of lift may vary from the actual frequency of the AEs associated with each treatment category.

## 5. Discussion and Conclusions

Adverse events present significant obstacles to the appropriate treatment of BC patients and have a negative impact on the quality of life. Disparities in AEs and their effects on the subsequent treatment of AA patients who experience them likely contribute to differences in the quality and quantity of care and resulting outcomes [61,62]. Relevant, specific, practical data on the impact of race and venue of treatment on the likelihood of AEs in the real world would contribute greatly to improved care but are not available. AEs reported in package inserts obtained either during clinical trials testing for drug registration or from post-marketing reporting are presented in no particular order and do not provide insight into the impact of race, stage, or venue of care on their likelihood of occurrence in racial subsets, stage of disease, or venue of administration. While some population-based studies report the likelihood of specific AEs in BC therapy, they do not provide these predictors [57,58]. Also, many studies on the racial impact on AEs are small and lack predictive value [17,18,59,63].

To this end, we undertook the TAR Mining approach using the very large S-M dataset to generate high-lift TR/AE associations in patients parsed by race, stage, and care venue. Association rule mining is a data-driven process where a pattern is derived based on the available data while making no assumption about the extracted pattern. Our comprehensive lists of temporal associations between drug categories and AEs for BC patients of each race with either localized disease or recurrent or metastatic disease who receive treatment in an Inst. OP or PPO settings will provide relevant clinical data. Our data demonstrated significant differences in AEs associated with TRs by race and venue. They will inform oncologists of greater-than-expected associations of certain AE categories with drug categories administered in these specific subsets in order of likelihood. They will serve as an additional point of reference for cancer caregivers to allow quick identification of relevant toxicity risks in similar patients having received the anticipated treatments and potentially improve the balance between benefit and risk.

Our study has several limitations. Firstly, the data do not differentiate between low to moderate grades 1–2 AEs and severe grades 3–4 AEs. Such data require the use of primary electronic medical records for assessment. To address this limitation, in future investigations with the S-M datasets, we will define severe AEs as those that result in admissions to emergency departments or inpatient hospital units or death [57,58]. Also, drugs from more than one category are administered together, and according to the algorithm, the AE is attributed to both. This is compatible with clinical trials attribution of AEs to drugs given together. To address this challenge, future investigations will use datasets where the ICD-10 coding system with more than 80,000 symptom codes in addition to the more than 14,000 symptom codes in the ICD-9 system used during the majority of the years spanning the dataset we analyzed currently. This will permit more specific AE associations with specific TRs. Another factor to be considered with the S-M data is that our study patient population was enrolled for age 65 years or older, which is older than the general population. To place the data in context and overcome this limitation, our future investigations will involve other datasets more representative of the age of the general population. It is also important to point out that association does not necessarily imply a causal effect. The method of overcoming this potential limitation is to conduct the study in several different datasets from different sources and corroborate the results for consistency. Despite these limitations, this approach is the first to provide clear distinctions in anticipated AEs based on race, stage, and venue, and these discovered patterns can be used to generate hypotheses for follow-up investigations.

## Figures and Tables

**Table 1:** Inclusion criteria.

Private Practice Office (PPO)		
PLCSRVC		11 = Office
**Institutional Outpatient (Inst. OP)**		
CLM-TYPE	Claim type	40 = Outpatient claim, 41 = Outpatient ‘Full-Encounter’ claim (available in the National Medicare Utilization Database (NMUD)), 42 = Outpatient ‘Abbreviated—Encounter’, or 71 = record identification code (RIC) O local carrier non-durable medical equipment (DMEPOS) Claim
OPSRVTYP	Outpatient service type	3 = elective
FAC_TYP	Facility type	1 = hospital or 2 = skilled nursing facility
PLCSRVC	Place of Service	13 = assisted living facility, 22 = outpatient hospital, 26 = military treatment facility, 31 = skilled nursing facility, 32 = nursing facility, 50 = federally qualified health center, 71 = state or local public health clinic, or 72 = rural health clinic.

**Table 2:** Patient characteristics.

	W			AA			
	Patients (% in Stage Group)	Age ± SD	Comorbidity Index ± SD	Patients (% in Stage Group)	AA/(W + AA) (%)	Age ± SD	Comorbidity Index ± SD
Stage I–III							
Inst OP.	196,768 (94.70%)	75.4 ± 7.2	2.9 ± 3.1	15,857 (91.45%)	7.46%	74.9 ± 7.2	3.3 ± 3.3
PP Ofc	202,090 (94.72%)	75.3 ± 7.2	2.6 ± 2.8	15,930 (91.76%)	7.31%	74.9 ± 7.2	2.9 ± 3.0
Stage IV							
Inst OP.	11,021 (5.30%)	76.3 ± 7.5	1.8 ± 2.8	1483 (8.55%)	11.86%	75.2 ± 7.4	1.8 ± 2.8
PP Ofc	11,261 (5.28%)	76.4 ± 7.5	1.6 ± 2.5	1431 (8.24%)	11.27%	75.4 ± 7.4	1.7 ± 2.6

**Table 3:** Example of comparisons of predicted and actual TR/AE associations in Stages I–III Inst. OP W and AA patients.

Actual TR-Associated AEs	TAR Mining	Actual TR-Associated AEs	TAR Mining
**TR** Most frequent AEs	AE category	**TR** Most frequent AEs	AE category
**Taxanes** (*n* = 13,519)	**Taxanes**	**Taxanes** (*n* = 1766)	**Taxanes**
Nausea/vomiting	Anemia	Nausea/vomiting	Anemia
Weakness/malaise	Pulmonary embolism	Neutropenia/Leukop.	Electrolyte abnormalities
Neutropenia/Leukop.	Neutropenia/leukopenia	Weakness/malaise	Neutropenia/leukopenia
Respiratory sympt.	Diarrhea	Anemias	Constipation
Electrolyte abn.	Electrolyte abnormalities	Electrolyte abn.	Respiratory symptoms
Anemias	Thrombophilia	Respiratory sympt.	Infection/fever
Diarrhea	Mucositis	Diarrhea	Weakness/malaise
Infection/fever	Weakness/malaise	Infection/fever	Nausea/vomiting
Constipation	Weight loss/malnutrition	Constipation	Diarrhea
Thrombophilia	Nausea/vomiting	Thrombophilia	Thrombophilia
Pulm. Embolus	Infection/fever	Weight loss/ malnut.	Mucositis
Total tallied	Constipation	Total tallied	Weight loss/malnutrition
	Respiratory symptoms		
	Rash		
**Her2 Ab** (*n* = 10,000)	**Her2 Ab**	**Her2 Ab** (*n* = 1195)	**Her2 Ab**
Weakness/malaise	Anemia	Weakness/malaise	Neutropenia/leukopenia
Nausea/vomiting	Neutropenia/leukopenia	Nausea/vomiting	Pulmonary embolism
Neutropenia/Leukop.	Electrolyte abn.	Neutropenia/Leukop	Weakness/malaise
Respiratory sympt.	Nausea/vomiting	Respiratory sympt.	Nausea/vomiting
Anemias	Diarrhea	Anemias	Electrolyte abnormalities
Diarrhea	Respiratory symptoms	Infection/fever	Thrombophilia
Infection/fever	Weakness/malaise	Thrombophilia	Infection/fever
Electrolyte abn.	Constipation	Diarrhea	Respiratory symptoms
Thrombophilia	Thrombophilia	Electrolyte abn.	Diarrhea
Constipation	Rash	Pulm. Embolus	Rash
Pulm. Embolus	Weight loss/malnut.	Constipation	
Weight loss/ malnut.	Infection/fever	Weight loss/ malnut.	
Skin rashes	Pulmonary embolism		
Total tallied	Mucositis	Total tallied	

## Data Availability

The original contributions presented in the study are included in the article/Supplementary Material. The original patient data were obtained from SEER-Medicare and are available from SEER-Medicare by request after a review process.

## Supplementary Materials

The following supporting information can be downloaded at: https://www.mdpi.com/article/10.3390/biomedicines12061213/s1, Table S1: Association of Treatment and Adverse events categories defined by lift (≥2) in the Institutional Outpatient setting for Stage I–III White patients and Stage I–III AA patients.; Table S2: Association of Treatment and Adverse events categories defined by lift (≥2) in the Institutional Outpatient setting for Stage IV White patients and Stage IV AA patients.; Table S3: Association of Treatment and Adverse events categories defined by lift (≥2) in the Private Practice (PP) Office setting for Stage I–III White patients and Stage I–III AA patients.; Table S4: Association of Treatment and Adverse events categories defined by lift (≥2) in the Private Practice (PP) Office setting for Stage IV White patients and Stage IV AA patients.

## Abbreviations

AA patients	African American patients
AE	adverse event
BC	breast cancer
CCFlag	Chronic Conditions Flag
CLM_TYPE	claim type
CMS	Centers for Medicare & Medicaid Services
Confidence(X→Y)	the probability that transactions Y appear in a dataset, given that X appears in the dataset
Dirsup	direct support
ER	Estrogen Receptor
FAC_TYPE	Facility type
FP-Growth,	database in the form of a tree called a frequent pattern tree (FP tree)
Her2	Human Epidermal Growth Factor Receptor 2
Her2-DM1	ado-trastuzumab emtansine
HCPCS drug J codes	Healthcare Common Procedure Coding System standardized method for reporting non-oral medications
HIV	Human Immunodeficiency Virus
HMO	Health Maintenance Organization
ICD-9 codes	International Classification of Diseases, Ninth Revision
Inst. OP	Institutional Outpatient
Lift(X→Y)	the degree of correlation between X and Y (e.g., TR and AE)
MEDPAR	Medicare Provider Analysis and Review
Melt transformation	a tool for reshaping data, turning columns into rows, particularly useful when tidying up wide datasets for analysis.
mTOR	mammalian target of rapamycin
minSupp	minimum support value
NCH	National Claims History
NCI	National Cancer Institute
OPSRVTYP	Outpatient service type
OMOP	Observational Medical Outcomes Partnership
Outpatient	Outpatient Claims
Part D	Prescription Drug Event Claims
PEDSF	Patient Entitlement and Diagnosis Summary File
PFP	parallel version FPGrowth which distributes the work of growing FP-trees based on the suffixes of transactions, resulting in a scalable implementation.
PLCSRVC	Place of Service
PPO	Private Practice Office
PR	Progesterone Receptor
SEER	Surveillance, Epidemiology, and End Results
S-M	SEER-Medicare
Support(X)	rhe probability that transaction X appears in a dataset
TAR mining	temporal association rule mining
TB	tuberculosis
TR	treatment
Trans	temporal transactions
Ս	union of two sets
Ո	intersection of two sets
VEGF	Vascular Endothelial Growth Gactor
W patients	White patients
