# Supplementary material for: Temporal Association Rule Mining: Race-Based Patterns of Treatment-Adverse Events in Breast Cancer Patients Using SEER–Medicare Dataset
Source: Biomedicines. 2024 May 29;12(6):1213. doi: 10.3390/biomedicines12061213 (PMC11200891; doi:10.3390/biomedicines12061213)
Supplement: Supplementary file 1 [file biomedicines-12-01213-s001.zip › biomedicines-2946541-supplementary.pdf]

SUPPLEMENTARY TABLES

Table S1. Association of Treatment and Adverse events categories defined by lift (≥ 2) in the Institutional Outpatient setting for Stage I-III White patients and Stage I-III AA patients.

**Institutional Outpatient**				
Stage I-III				
	White patients		AA Patients	
Treatment category	lift	AE category	lift	AE category
Taxanes	6.5	Anemia	6.1	Anemia
	4.8	Pulmonary embolism	4.9	Electrolyte abnormalities
	4.6	Neutropenia/leukopenia	4.4	Neutropenia/leukopenia
	4.4	Diarrhea	3.8	Constipation
	4.2	Electrolyte abnormalities	3.5 (4.8)*	Respiratory symptoms
	3.9	Thrombophilia	3.5 (4.4)*	Infection/fever
	3.8	Mucositis	3.2	Weakness/malaise
	3.5 (4.5)*	Weakness/malaise	3.1	Nausea/vomiting
	3.4	Weight loss/malnutrition	3.1	Diarrhea
	3.2	Nausea/vomiting	3.0	Thrombophilia
	3.2	Infection/fever	3.0	Mucositis
	3.2	Constipation	2.1	Weight loss/malnutrition
	2.7	Respiratory symptoms		
	2.2	Rash		
Her2 Ab	6.3	Anemia	5.4	Neutropenia/leukopenia
	6.0	Neutropenia/leukopenia	5.0	Pulmonary embolism
	5.0	Electrolyte abnormalities	4.0	Weakness/malaise
	4.6 (5.9)*	Nausea/vomiting	3.5 (4.2)*	Nausea/vomiting
	3.0	Diarrhea	3.2	Electrolyte abnormalities
	2.9	Respiratory symptoms	3.1	Thrombophilia
	2.9	Weakness/malaise	2.7	Infection/fever
	2.8	Constipation	2.6	Respiratory symptoms
	2.8	Thrombophilia	2.5	Diarrhea
	2.7	Rash	2.5	Rash
	2.6	Weight loss/malnutrition		
	2.4	Infection/fever		
	2.4	Pulmonary embolism		
	2.3	Mucositis		
Bisphosphonates	6.1	Neutropenia/leukopenia	5.2	Weakness/malaise
	5.4	Weakness/malaise	5.0	Nausea/vomiting
	5.0	Rash	4.0	Anemia
	4.5	Nausea/vomiting	3.0	Thrombophilia
	3.8	Thrombophilia	2.5	Respiratory symptoms
	3.7	Electrolyte abnormalities	2.5	Constipation
	3.6 (5.1)*	Pulmonary embolism	2.4	Infection/fever
	3.4	Weight loss/malnutrition		
	3.2	Diarrhea		
	3.0	Infection/fever		
	2.4	Constipation		
	2.3	Respiratory symptoms		
	2.3	Mucositis		
Alkylating agents	4.1	Respiratory symptoms	5.3	Respiratory symptoms

Table S1.(continued)

**Institutional Outpatient**				
Stage I-III				
	White patients		AA Patients	
Treatment category	lift	AE category	lift	AE category
Anthracyclines	5.4	Nausea/vomiting	4.5	Nausea/vomiting
	3.9	Thrombophilia	3.6	Neutropenia/leukopenia
	3.8	Anemia	3.5	Anemia
	3.3	Weakness/malaise	3.2	Diarrhea
	3.1	Pulmonary embolism	3.1	Electrolyte abnormalities
	3.0 (4.0)*	Rash	2.8	Respiratory symptoms
	2.8	Neutropenia/leukopenia	2.5	Weakness/malaise
	2.7	Infection/fever	2.2	Infection/fever
	2.5	Mucositis	2.2	Constipation
	2.4	Constipation	2.2	Thrombophilia
	2.3	Diarrhea		
	2.3	Electrolyte abnormalities		
	2.2	Respiratory symptoms		
	2.1	Weight loss/malnutrition		
Pyrimidine analogs	4.4	Constipation	6.5	Neutropenia/leukopenia
	3.9	Electrolyte abnormalities	5.4	Anemia
	3.8	Neutropenia/leukopenia	4.2 (4.8)*	Nausea/vomiting
	3.7	Thrombophilia	4.0 (5.2)*	Weakness/malaise
	3.4 (4.6)*	Weakness/malaise	3.0	Diarrhea
	3.2	Weight loss/malnutrition	2.9	Respiratory symptoms
	3.2	Pulmonary embolism	2.6	Infection/fever
	3.0	Diarrhea	2.3	Weight loss/malnutrition
	3.0	Respiratory symptoms	2.2	Constipation
	2.4	Infection/fever	2.1	Thrombophilia
	2.2	Nausea/vomiting		
	2.0	Rash		
Antiestrogens	6.4	Weakness/malaise	5.7	Weakness/malaise
	5.4	Diarrhea	2.5	Infection/fever
	5.0	Rash	2.5	Constipation
	4.7	Nausea/vomiting	2.4	Respiratory symptoms
	4.0	Electrolyte abnormalities	2.2	Thrombophilia
	3.9	Weight loss/malnutrition	2.0	Electrolyte abnormalities
	3.2	Constipation		
	3.1 (4.0)*	Pulmonary embolism		
	2.6	Thrombophilia		
	2.5	Infection/fever		
	2.5	Respiratory symptoms		
Platinum compounds	6.0	Weight loss/malnutrition	5.2	Nausea/vomiting
	4.3	Pulmonary embolism	4.0	Weakness/malaise
	4.2	Anemia	3.5	Anemia
	3.5	Weakness/malaise	3.4	Diarrhea
	3.4	Constipation	2.6	Thrombophilia
	3.3 (4.6)*	Nausea/vomiting	2.5	Infection/fever
	3.2	Neutropenia/leukopenia	2.5	Respiratory symptoms
	3.2	Thrombophilia	2.2	Neutropenia/leukopenia
	2.7	Electrolyte abnormalities	2.2	Electrolyte abnormalities
	2.5	Infection/fever	2.2	Constipation
	2.5	Mucositis		
	2.5	Respiratory symptoms		
	2.4	Diarrhea		

Table S1.(continued)

**Institutional Outpatient**				
Stage I-III				
	White patients		AA Patients	
Treatment category	lift	AE category	lift	AE category
Vinca alkaloids	5.5	Pulmonary embolism	4.7 (5.8)*	Nausea/vomiting
	4.2	Diarrhea	4.0	Anemia
	4.0	Weight loss/malnutrition	3.0	Weight loss/malnutrition
	3.9 (5.0)*	Nausea/vomiting	2.9	Respiratory symptoms
	3.8	Neutropenia/leukopenia	2.5	Weakness/malaise
	3.3	Weakness/malaise		
	2.8	Respiratory symptoms		
	2.6	Constipation		
	2.5	Infection/fever		
Folate analogs	6.4	Respiratory symptoms	2.0	Respiratory symptoms
VEGF Inhibitors	5.3	Neutropenia/leukopenia	4.0	Neutropenia/leukopenia
	4.6 (6.1)*	Nausea/vomiting	4.0	Electrolyte abnormalities
	4.0	Electrolyte abnormalities	3.0	Respiratory symptoms
	3.3	Diarrhea	2.9	Weakness/malaise
	3.2	Anemia	2.8	Thrombophilia
	3.0	Constipation	2.0	Diarrhea
	2.9	Respiratory symptoms		
	2.3	Infection/fever		
	2.2	Weakness/malaise		
Her2-DM1	4.0	Electrolyte abnormalities	2.9	Weakness/malaise
	4.0	Weakness/malaise	2.5	Nausea/vomiting
	2.5	Constipation		
	2.4	Respiratory symptoms		
	2.0	Neutropenia/leukopenia		
	2.0	Thrombophilia		
mTOR Inhibitors	2.5	Anemia		
	2.5	Respiratory symptoms		
	2.4	Weakness/malaise		
Somatostatin Analogs	2.0	Infection/fever		
	2.0	Weakness/malaise		

*Lift values discovered when temporal associations were determined at lift of $\geq$ 4.0

Table S2. Association of Treatment and Adverse events categories defined by lift (≥ 2) in the Institutional Outpatient setting for Stage IV White patients and Stage IV AA patients.

**Institutional Outpatient**				
Stage IV				
Treatment category	White patients		AA Patients	
	lift	AE category	lift	AE category
Taxanes	5.8	Anemia	6.3	Neutropenia/leukopenia
	5.3	Neutropenia/leukopenia	5.5	Pulmonary embolism
	4.7	Thrombophilia	5.0	Thrombophilia
	4.2 (5.0)*	Infection/fever	4.8	Electrolyte abnormalities
	4.1	Diarrhea	4.5	Respiratory symptoms
	3.9 (4.8)*	Weakness/malaise	4.3	Constipation
	3.8	Electrolyte abnormalities	3.9	Nausea/vomiting
	3.8	Weight loss/malnutrition	3.8 (4.6)^	Weakness/malaise
	3.3	Constipation		
	3.3	Pulmonary embolism		
	2.9	Respiratory symptoms		
	2.8	Nausea/vomiting		
Her2 Ab	6.0	Thrombophilia	5.3	Anemia
	5.8	Anemia	5.0	Nausea/vomiting
	4.5 (5.2)*	Infection/fever	4.6 (5.5)*	Respiratory symptoms
	4.0	Weakness/malaise	4.0	Neutropenia/leukopenia
	3.6 (5.0)*	Nausea/vomiting	2.6	Weakness/malaise
	3.5 (5.5)*	Pulmonary embolism		
	3.1	Constipation		
	3.0	Neutropenia/leukopenia		
	2.5	Electrolyte abnormalities		
	2.5	Respiratory symptoms		
	2.5	Mucositis		
	2.4	Diarrhea		
	2.2	Weight loss/malnutrition		
Bisphosphonates	6.3	Neutropenia/leukopenia	5.5	Nausea/vomiting
	5.7	Thrombophilia	3.4 (4.0)*	Electrolyte abnormalities
	5.2	Anemia	3.4 (4.0)*	Infection/fever
	4.3 (6.5)*	Pulmonary embolism	3.4 (4.0)*	Respiratory symptoms
	4.2	Weight loss/malnutrition	3.3	Constipation
	3.5	Electrolyte abnormalities	3.2	Neutropenia/leukopenia
	3.1	Diarrhea	3.2	Weakness/malaise
	2.8	Infection/fever	3.2	Weight loss/malnutrition
	2.7	Nausea/vomiting	2.5	Pulmonary embolism
	2.7	Constipation		
	2.4	Respiratory symptoms		
Alkylating agents	5.0	Respiratory symptoms		
Anthracyclines	5.0	Thrombophilia	5.0	Neutropenia/leukopenia
	4.8	Weakness/malaise	3.5 (5.0)*	Nausea/vomiting
	3.2	Electrolyte abnormalities	3.0	Weakness/malaise
	2.7	Respiratory symptoms	2.5	Electrolyte abnormalities
	2.5	Diarrhea		
	2.5	Pulmonary embolism		
	2.4	Neutropenia/leukopenia		
	2.4	Infection/fever		
	2.0	Constipation		

Table S2. (continued)

Institutional Outpatient				
Stage IV				
	White patients		AA Patients	
Treatment category	lift	AE category	lift	AE category
Pyrimidine analogs	5.6	Nausea/vomiting	3.0	Neutropenia/leukopenia
	4.5	Constipation	2.7	Respiratory symptoms
	3.9	Neutropenia/leukopenia	2.5	Constipation
	3.5	Electrolyte abnormalities	2.5	Weakness/malaise
	3.5	Infection/fever		
	3.5	Weakness/malaise		
	3.5	Weight loss/malnutrition		
	3.0	Respiratory symptoms		
	3.0	Diarrhea		
Antiestrogens	5.6	Anemia	3.4	Respiratory symptoms
	4.2	Diarrhea	3.4 (4.5)*	Weakness/malaise
	4.0	Thrombophilia	3.0	Nausea/vomiting
	3.9	Constipation	2.5	Pulmonary embolism
	3.8 (5.6)*	Weakness/malaise	2.3	Thrombophilia
	3.5	Pulmonary embolism		
	3.2 (4.0)*	Respiratory symptoms		
	3.0	Electrolyte abnormalities		
	3.0	Weight loss/malnutrition		
	2.4	Infection/fever		
Platinum compounds	3.3	Constipation	4.0	Nausea/vomiting
	3.2	Electrolyte abnormalities	3.2	Neutropenia/leukopenia
	3.0	Anemia	2.0	Weakness/malaise
	2.9	Weakness/malaise		
	2.6	Neutropenia/leukopenia		
	2.4	Infection/fever		
	2.2	Diarrhea		
	2.1	Respiratory symptoms		
	2.0	Thrombophilia		
Vinca alkaloids	6.5	Thrombophilia	6.0	Neutropenia/leukopenia
	5.3	Nausea/vomiting	2.6	Respiratory symptoms
	3.5	Electrolyte abnormalities	2.5	Nausea/vomiting
	3.5	Weakness/malaise	2.5	Weakness/malaise
	3.4 (4.0)*	Infection/fever		
	3.0	Respiratory symptoms		
	2.8	Neutropenia/leukopenia		
VEGF Inhibitors	3.5	Anemia	2.0	Weakness/malaise
	3.0	Nausea/vomiting		
	2.5	Diarrhea		
	2.5	Constipation		
	2.2	Respiratory symptoms		
	2.2	Weakness/malaise		
	2.0	Electrolyte abnormalities		
Her2-DM1	2.8	Nausea/vomiting		
	2.5	Neutropenia/leukopenia		
Interleukins	5.3	Anemia		

*Lift values discovered when temporal associations were determined at lift of $\geq$ 4.0

Table S3. Association of Treatment and Adverse events categories defined by lift (> 2) in the Private Practice (PP) Office setting for Stage I-III White patients and Stage I-III AA patients.

**Private Practice Office**				
Stage I-III				
	White patients		AA Patients	
Treatment category	lift	AE category	lift	AE category
Taxanes	6.4	Nausea/vomiting	5.9	Nausea/vomiting
	6.1	Electrolyte abnormalities	4.7 (5.4)*	Respiratory symptoms
	4.1	Weakness/malaise	4.3	Pulmonary embolism
	3.9	Infection/fever	4.0 (4.8)*	Infection/fever
	3.5	Thrombophilia	3.6	Thrombophilia
	3.4 (4.5)*	Respiratory symptoms	3.2	Constipation
	3.2	Pulmonary embolism	2.5	Mucositis
	3.0	Diarrhea	2.3	Weakness/malaise
	2.6	Constipation	2.2	Weight loss/malnutrition
	2.5	Mucositis	2.0	Diarrhea
	2.4	Neutropenia/leukopenia	2.0	Rash
Her2 Ab	5.1	Nausea/Vomiting	6.5	Neutropenia/leukopenia
	4.3	Neutropenia/leukopenia	6.5	Pulmonary embolism
	3.8 (4.0)*	Respiratory symptoms	6.0	Nausea/vomiting
	3.3	Weight loss/malnutrition	4.6	Respiratory symptoms
	3.0	Pulmonary embolism	3.3	Diarrhea
	2.6	Weakness/malaise	2.7	Constipation
	2.3	Diarrhea	2.5	Weakness/malaise
	2.2	Thrombophilia	2.1	Thrombophilia
	2.2	Mucositis	2.0	Weight loss/malnutrition
	2.2	Constipation		
	2.0	Neurpathy		
Bisphosphonates	5.8	Infection/fever	6.5	Anemia
	4.4	Neutropenia/leukopenia	6.2	Infection/fever
	3.7	Weakness/malaise	5.0	Weakness/malaise
	3.7 (4.4)*	Pulmonary embolism	3.4 (4.0)*	Pulmonary embolism
	3.2	Respiratory symptoms	3.0	Diarrhea
	2.9	Nausea/vomiting	2.7	Nausea/vomiting
	2.8	Constipation	2.7	Constipation
	2.5	Diarrhea	2.3	Respiratory symptoms
	2.3	Mucositis	2.1	Thrombophilia
	2.1	Weight loss/malnutrition	2.0	Weight loss/malnutrition
	2.1	Thrombophilia		
Alkylating agents	5.5	Respiratory symptoms	6.2	Neutropenia/leukopenia
	5.0	Infection/fever	6.0	Respiratory symptoms
	5.0	Rash		
Anthracyclines	4.9	Electrolyte abnormalities	4.6	Anemia
	4.8	Infection/fever	3.6	Infection/fever
	4.4	Anemia	3.2	Neutropenia/leukopenia
	4.4 (5.5)*	Pulmonary embolism	2.9	Weakness/malaise
	3.0	Respiratory symptoms	2.4	Respiratory symptoms
	2.6	Weakness/malaise		
	2.3	Mucositis		
	2.2	Neutropenia/leukopenia		
	2.2	Constipation		
	2.2	Diarrhea		
	2.2	Thrombophilia		
	2.1	Nausea/vomiting		
	2.1	Weight loss/malnutrition		

Table S3.(continued)

Private Practice Office				
Stage I-III				
	White patients		AA Patients	
Treatment category	lift	AE category	lift	AE category
Pyrimidine analogs	4.7 (5.6)*	Infection/fever	6.0	Thrombophilia
	4.2 (4.4)*	Rash	4.2 (6.3)*	Respiratory symptoms
	4.0	Mucositis	4.2	Weakness/malaise
	3.9	Pulmonary embolism	3.4 (4.0)*	Pulmonary embolism
	3.4	Weakness/malaise	3.3	Nausea/vomiting
	3.4	Thrombophilia	3.0	Infection/fever
	3.3	Weight loss/malnutrition	2.9	Neutropenia/leukopenia
	3.1	Nausea/vomiting		
	2.8	Respiratory symptoms		
	2.4	Diarrhea		
	2.3	Neutropenia/leukopenia		
	2.0	Constipation		
Antiestrogens	6.1	Weakness/malaise	5.2	Electrolyte abnormalities
	4.9	Neutropenia/leukopenia	4.2	Nausea/vomiting
	4.7	Infection/fever	3.5	Pulmonary embolism
	4.5	Mucositis	2.8	Weakness/malaise
	3.5	Weight loss/malnutrition	2.5	Respiratory symptoms
	2.9	Nausea/vomiting		
	2.9	Thrombophilia		
	2.7	Respiratory symptoms		
	2.7	Pulmonary embolism		
	2.4	Constipation		
	2.1	Diarrhea		
Platinum compounds	5.7	Anemia	4.2	Electrolyte abnormalities
	5.2	Pulmonary embolism	3.6	Neutropenia/leukopenia
	3.3	Mucositis	3.5	Pulmonary embolism
	3.2	Respiratory symptoms	2.8	Nausea/vomiting
	3.0	Electrolyte abnormalities	2.8	Weakness/malaise
	2.9	Weakness/malaise	2.5	Respiratory symptoms
	2.8	Nausea/vomiting		
	2.8	Weight loss/malnutrition		
	2.5	Neutropenia/leukopenia		
	2.5	Thrombophilia		
	2.4	Constipation		
	2.2	Diarrhea		
Vinca alkaloids	4.5	Thrombophilia	5.3	Weakness/malaise
	3.7	Diarrhea	5.0	Pulmonary embolism
	3.4	Infection/fever	3.0	Infection/fever
	3.3	Weakness/malaise	2.5	Respiratory symptoms
	3.2	Weight loss/malnutrition	2.4	Neutropenia/leukopenia
	3.0 (4.0)*	Respiratory symptoms		
	2.4	Pulmonary embolism		
	2.3	Neutropenia/leukopenia		
	2.3	Constipation		
Folate analogs	6.6	Weakness/malaise	2.8	Infection/fever
	5.4	Respiratory symptoms	2.5	Mucositis
	4.7	Constipation		
	3.7 (4.6)*	Infection/fever		
	3.3 (4.1)*	Rash		
	2.2	Weight loss/malnutrition		

Table S3.(continued)

**Private Practice Office**				
Stage I-III				
	White patients		AA Patients	
Treatment category	lift	AE category	lift	AE category
VEGF Inhibitors	6.7 (6.2)*	Weakness/malaise	5.1	Anemia
	5.7	Nausea/vomiting	5.0	Electrolyte abnormalities
	5.2	Electrolyte abnormalities	3.5	Nausea/vomiting
	4.5 (6.0)*	Mucositis	3.4 (4.8)*	Infection/fever
	4.1	Anemia	3.4	Neutropenia/leukopenia
	3.0	Infection/fever		
	2.7	Neutropenia/leukopenia		
	2.4	Pulmonary embolism		
Her2-DM1	2.5	Electrolyte abnormalities	6.0	Weakness/malaise
	2.3	Nausea/vomiting		
	2.3	Weakness/malaise		
Cytotoxic Antibiotics	3.0	Rash		
	2.5	Respiratory symptoms		
	2.0	Thrombophilia		
EGFR Inhibitors	2.0	Nausea/Vomiting		
Proteosome inhibitors	3.8	Anemia		
	3.4	Nausea/vomiting		
	2.9	Weakness/malaise		
	2.5	Respiratory symptoms		
	2.5	Neutropenia/leukopenia		
Interleukins	4.0	Electrolyte abnormalities		
	3.3	Nausea/vomiting		
	2.0	Weakness/malaise		
Somatostatin Analogs	3.5	Anemia		
	2.8	Weakness/Malaise		
	2.5	Infection/fever		
	2.0	Nausea/Vomiting		

*Lift values discovered when temporal associations were determined at lift of $\geq$ 4.0

Table S4. Association of Treatment and Adverse events categories defined by lift ($\geq$ 2) in the Private Practice (PP) Office setting for Stage IV White patients and Stage IV AA patients.

**Private Practice Office**				
Stage IV				
	White patients		AA Patients	
Treatment category	lift	AE category	lift	AE category
Taxanes	5.0 (6.2)*	Weakness/malaise	5.4	Nausea/vomiting
	4.6	Thrombophilia	5.0	Weakness/malaise
	4.2 (5.8)*	Infection/fever	3.3	Respiratory symptoms
	3.0	Pulmonary Embolism	2.3	Neutropenia/leukopenia
	2.9	Constipation	2.0	Thrombophilia
	2.7	Weight loss/malnutrition		
	2.6	Diarrhea		
	2.5	Respiratory symptoms		
	2.4	Neutropenia/Leukopenia		
Her2 Ab	6.2	Anemia	3.8	Thrombophilia
	5.2	Infection/fever	2.6	Neutropenia/leukopenia
	5.1	Nausea/Vomiting	2.5	Weight loss/malnutrition
	3.7	Respiratory symptoms	2.1	Weakness/malaise
	3.0	Constipation		
	3.0	Weakness/malaise		
	2.9	Neutropenia/Leukopenia		
	2.8	Weight loss/malnutrition		
	2.7	Thrombophilia		
	2.3	Diarrhea		
	2.2	Pulmonary Embolism		
Bisphosphonates	6.1	Thrombophilia	5.5	Weakness/malaise
	5.2	Infection/fever	4.2	Neutropenia/leukopenia
	4.1	Neutropenia/Leukopenia	3.7	Nausea/vomiting
	3.8	Weight loss/malnutrition	3.0	Thrombophilia
	3.4	Nausea/Vomiting	2.4	Infection/fever
	2.3	Diarrhea	2.2	Respiratory symptoms
	2.3	Constipation		
	2.2	Respiratory symptoms		
Alkylating Agents	3.7	Thrombophilia		
	2.8	Infection/fever		
	2.0	Respiratory symptoms		
Anthracyclines	6.3	Anemia	4.6	Anemia
	3.0	Thrombophilia	3.8	Nausea/vomiting
	2.8	Neutropenia/Leukopenia	3.0	Neutropenia/leukopenia
	2.8	Respiratory symptoms	2.7	Electrolyte abnormalities
	2.8	Weakness/malaise	2.5	Weakness/malaise
	2.4	Infection/fever		
	2.4	Nausea/Vomiting		
	2.3	Constipation		
	2.0	Diarrhea		
	2.0	Weight loss/malnutrition		
Pyrimidine Analogs	6.3	Nausea/Vomiting	4.5	Neutropenia/leukopenia
	4.2	Diarrhea	2.5	Respiratory symptoms
	3.5	Weakness/malaise		
	3.2	Infection/fever		
	2.7	Constipation		
	2.7	Respiratory symptoms		

Table S4. (continued)

**Private Practice Office**				
Stage IV				
	White patients		AA Patients	
Treatment category	lift	AE category	lift	AE category
Antiestrogens	4.5 (6.6)*	Weakness/malaise	3.5	Thrombophilia
	4.2	Infection/fever	3.2	Infection/fever
	4.2	Respiratory symptoms	2.9	Respiratory symptoms
	3.8	Rash	2.0	Weakness/malaise
	3.2	Constipation		
	2.4	Nausea/Vomiting		
	2.4	Thrombophilia		
	2.1	Diarrhea		
	2.0	Pulmonary Embolism		
Platinum Compounds	6.0	Anemia	5.0	Weakness/malaise
	4.3	Respiratory symptoms	4.7	Electrolyte abnormalities
	3.9	Electrolyte abnormalities	3.8	Nausea/vomiting
	3.9	Infection/fever	3.1	Neutropenia/leukopenia
	3.3	Pulmonary Embolism		
	2.9	Nausea/Vomiting		
	2.9	Neutropenia/Leukopenia		
	2.2	Weakness/malaise		
	2.0	Weight loss/malnutrition		
Vinca Alkaloids	5.5	Electrolyte abnormalities		
	5.4	Weakness/malaise		
	4.3	Infection/fever		
	4.2	Constipation		
	3.2	Neutropenia/Leukopenia		
	2.7	Respiratory symptoms		
Folate Analogs	5.5	Neutropenia/Leukopenia		
	2.0	Diarrhea		
VEGF Inhibitors	5.3	Nausea/Vomiting	3.0	Anemia
	4.8	Electrolyte abnormalities		
	4.5	Respiratory symptoms		
	3.2	Anemia		
	3.1	Infection/fever		
	2.6	Weakness/malaise		
	2.5	Neutropenia/Leukopenia		
	2.3	Diarrhea		
Her2-DM1	2.9	Nausea/Vomiting		
mTOR Inhibitors	5.0	Constipation		
	2.7	Thrombophilia		
	2.5	Weakness/malaise		
Cytotoxic Antibiotics	2.8	Nausea/Vomiting		

*Lift values discovered when temporal associations were determined at lift of $\geq$ 4.0
